# Invasive Haemophilus influenzae type b disease in elderly nursing home residents: two related cases.

**DOI:** 10.3201/eid0302.970212

**Published:** 1997

**Authors:** T C Heath, M C Hewitt, B Jalaludin, C Roberts, A G Capon, P Jelfs, G L Gilbert

**Affiliations:** Western Sector Public Health Unit, North Parramatta, New South Wales.

## Abstract

We investigated two fatal cases of invasive Haemophilus influenzae type b (Hib) infection in a community nursing home in western Sydney, Australia. Two elderly women had lived in the same room, and the onset of their illness was 5 days apart. Hib isolates from blood cultures showed identical profiles by pulsed field gel electrophoresis. These findings suggest that Hib infection was transmitted within this nursing home. Serious Hib disease may be underrecognized in this setting. Continued surveillance and serotyping of invasive H. influenzae disease is essential for identifying groups at increasing risk that may benefit from immunization against Hib.


### Invasive *Haemophilus influenzae* type b Disease in Elderly Nursing Home Residents: Two Related Cases

We investigated two fatal cases of invasive *Haemophilus influenzae* type b (Hib) infection in a community nursing home in western Sydney, Australia. Two elderly women had lived in the same room, and the onset of their illness was 5 days apart. Hib isolates from blood cultures showed identical profiles by pulsed field gel electrophoresis. These findings suggest that Hib infection was transmitted within this nursing home. Serious Hib disease may be underrecognized in this setting. Continued surveillance and serotyping of invasive *H. influenzae* disease is essential for identifying groups at increasing risk that may benefit from immunization against Hib.

After the introduction of routine childhood immunization against *Haemophilus influenzae* type b (Hib), the incidence of invasive Hib disease fell dramatically in several countries, including Australia (1-3). This decline has been most evident among children aged less than 5 years. Meanwhile, serious Hib infections, such as pneumonia and epiglottitis, have been increasingly recognized among elderly, debilitated, and immunosuppressed adults (4-6). However, clusters of Hib infection have rarely been reported in adults. To examine their relatedness, we investigated two fatal cases of Hib septicemia in a community nursing home.

### Case Reports

### Case 1

In June 1996, a 71-year-old female nursing home resident was hospitalized after 3 days of unproductive cough, fevers, confusion, and increasing dyspnea. She had a history of cerebrovascular disease, muscular dystrophy, and congestive heart failure and was confined to a wheelchair. She had clinical signs of left lower lobe pneumonia and was afebrile. A blood film was normal, but a chest X-ray showed extensive bilateral pulmonary infiltrates. Arterial blood gases showed severe hypoxia in room air ($PO_2$ 44 mm Hg, saturation 77%). Ceftriaxone 1 g and metronidazole 500 mg were given intravenously for suspected aspiration pneumonia, but the patient became severely hypotensive and died 8 hours after admission to the hospital. The following day, beta-lactamase-negative Hib was isolated from blood cultures collected before the patient died.

### Case 2

Two days after the first patient was hospitalized, an 80-year-old female resident of the same nursing home became ill with fever and sore throat. She had a history of chronic airflow limitation, mild renal impairment, and dementia. Her sore throat rapidly worsened, and the next day she was hospitalized with severe dysphagia. On examination, she was febrile (38.5°C) and hypotensive (90/60 mm Hg) and had pretracheal cellulitis. No clinical evidence of pneumonia was observed, and a chest X-ray was normal. She was intubated, and extensive epiglottitis, laryngitis, and tracheitis were noted. Ceftriaxone 2 g, gentamicin 80 mg, flucloxacillin 1 g, and metronidazole 500 mg were administered intravenously. Inotropes were given for hypotension and acute renal failure, but the patient's condition deteriorated rapidly, and she died 9 hours after admission to the hospital. Beta-lactamase-negative Hib was subsequently isolated from blood cultures collected before the patient died.

### Investigation

For several months, these two women had occupied beds in the same six-bed room (the largest room) in the 44-bed nursing home. The home employed 37 nursing and ancillary staff. During 3 weeks before the investigation, 20 residents and several staff had been ill with bronchitis, for which many had received oral antibiotics. No sputum samples had been collected for culture from residents or staff. Two other elderly residents had died during the preceding 10 days, but their case notes did not document any fever or symptoms suggesting infection. On the first day of our investigation, two elderly residents were febrile. One woman had bilateral bronchopneumonia that was not responding to oral cefaclor and was hospitalized; blood and sputum cultures yielded no pathogens, and urine samples did not contain Hib capsular antigen. The other woman had been febrile for 1 week, with a cellulitic leg ulcer and pleuritic chest pain of uncertain etiology. Because of her other chronic illnesses she was treated palliatively and died 1 week later. A swab of her leg ulcer yielded group G streptococcus, blood cultures were negative, and sputum was not obtained.

Throat swabs were collected from all 81 residents and staff at the nursing home and cultured for Hib on bacitracin chocolate blood agar. We administered rifampicin chemoprophylaxis to all roommates of the index case patients and 28 staff who had been in regular close contact with the two case patients during the week before their illness. No new febrile illnesses occurred during 3 weeks of follow-up, and Hib was not isolated from any throat swab cultures. The Hib isolates from the first and second patients were examined by pulsed field gel electrophoresis and had identical profiles that were distinguishable from several epidemiologically unrelated isolates (Figure).

The relationship of these two fatal cases of Hib disease in time and place and the clonality of the isolates provide good evidence that transmission of Hib infection occurred in this nursing home. We were unable to demonstrate that Hib caused any of the associated reports of bronchitis and febrile illness among residents and staff, and our prevalence study did not show any asymptomatic Hib carriage. However, the survey was performed 10 days after the onset of the second case of septicemia following widespread use of antibiotics, and it may not have accurately reflected the prevalence of Hib carriage at the time these cases occurred. It is also difficult to evaluate whether chemoprophylaxis was a useful intervention in this setting.

To our knowledge, this is the first reported cluster of invasive Hib infection among adults in a community-based nursing home. Three other clusters of Hib infection in adults have been described: one occurred in a nursing home attached to a hospital, and two were nosocomial—including a cluster of bronchitic illness (7-9). Our report provides further evidence that Hib can cause clusters of serious disease among the elderly—at least in institutions. Given that diagnostic procedures are performed infrequently in many community nursing homes, Hib infection may be underrecognized in this setting.

We were especially interested in these two cases in adults as they were the only instances of

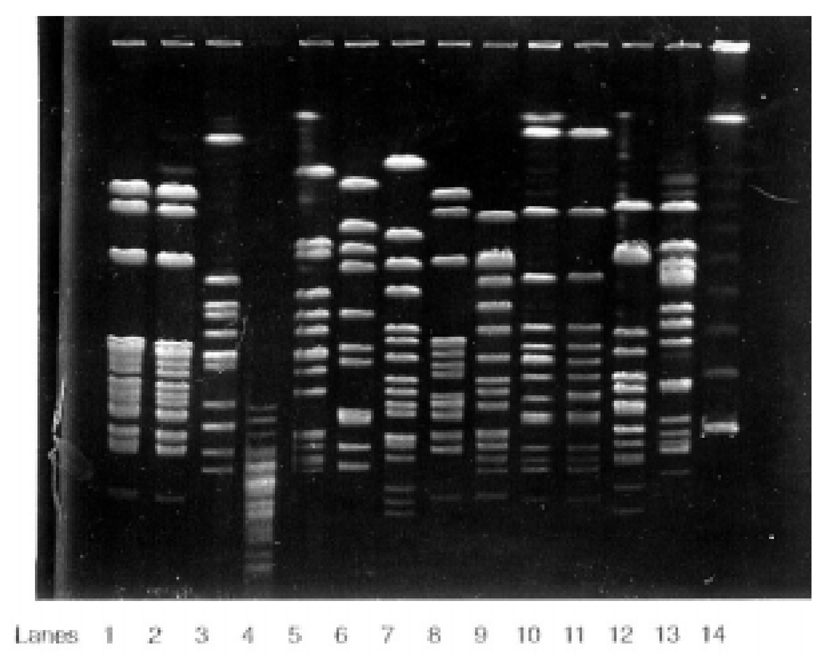

Figure. Pulsed field gel electrophoresis of *Haemophilus influenzae* type b (Hib) isolates from blood culture of two elderly nursing home residents (lanes 1 and 2) compared with epidemiologically unrelated *H. influenzae* isolates sent to our laboratory for typing: lane 3, laboratory Hib strain; lane 4, non-*H. influenzae*; lane 5 and 12, invasive nontypeable *H. influenzae*; lane 6-11, unrelated Hib isolates; lane 13, *H. influenzae* type a; lane 14, 1 kilobase molecular marker. The enzyme used for DNA digestion was *ApaI*.

invasive Hib disease reported to this Public Health Unit from its population base of one million persons over 12 months—a rate of 0.3 per 100,000 population aged 15 years and older. This pattern of notification differs markedly from that observed in western Sydney at the time childhood Hib vaccination was first introduced in mid-1992. In 1992, 27 of 30 notifications of invasive Hib disease in this area were of children aged less than 5 years, or 36.9 per 100,000 population (New South Wales Health Department, unpub. data). Although invasive Hib infection remains uncommon in adults in communities where childhood Hib vaccination is routine, the relative frequency of invasive Hib infection is increasing in older age groups.

Population-based studies of acute epiglottitis undertaken in northern California and Rhode Island have also shown that the incidence of childhood epiglottitis is falling rapidly since the introduction of Hib immunization, while the incidence of epiglottitis in adults remains steady or is increasing (10,11). Acute epiglottitis in children is usually caused by Hib, and the falling incidence of epiglottitis in this age group is explainable by immunization. In adults, most culture-positive cases of acute epiglottitis are also caused by Hib (10,12). However, the incidence of Hib-related epiglottitis in adults does not appear to be changing appreciably—at least in Rhode Island—and Hib does not account for the increasing incidence of epiglottitis observed in this age group (10). Indeed, acute epiglottitis is frequently culture negative in adults, so explaining its increasing incidence and ascertaining the etiologic agents in older age groups require further study (10,12).

Invasive Hib disease should continue to be monitored in all age groups after the introduction of childhood immunization against Hib. Evidence suggests that Hib immunization reduces the acquisition of Hib carriage in children, and reduced carriage among children may indirectly prevent Hib disease in adults (13). However, surveillance data in western Sydney and metropolitan Atlanta have not supported this hypothesis; rates of adult Hib disease are remaining approximately unchanged, despite dramatic reductions in childhood disease (5; New South Wales Health Department, unpub. data). Only by serotyping isolates of *H. influenzae* that cause invasive disease will it be possible to detect changing patterns of invasive Hib disease in adults during this era of childhood immunization. Serotype-specific surveillance of invasive *H. influenzae* disease is also needed to assess progress towards the elimination of childhood Hib disease. In addition, periodic cross-sectional serotyping studies of *H. influenzae* isolates causing noninvasive disease would help measure the effects of Hib immunization on the incidence of less serious forms of Hib infection. These surveillance methods will identify groups at increasing or underrecognized risk that may benefit from immunization.

### Acknowledgments

The authors thank Graham O'Donnell (Department of Microbiology, Westmead Hospital) and Marianne Kerr and Oanh Nguyen (Western Sector Public Health Unit). The Master of Applied Epidemiology Program is funded by the Commonwealth Department of Health and Family Services, Canberra.

**Timothy C. Heath,*† Moira C. Hewitt,†‡ Bin Jalaludin,* Christine Roberts,† Anthony G. Capon,* Peter Jelfs,§ and Gwendolyn L. Gilbert§**

*Western Sector Public Health Unit, North Parramatta, New South Wales; †Australian National University, Australian Capital Territory, Australia; ‡Department of Public Health and Community Medicine, Westmead, New South Wales; and §Institute of Clinical Pathology and Medical Research, Westmead, New South Wales

### References

1. Adams WG, Deaver KA, Cochi SL, Plikaytis BD, Zell ER, Broome CV, Wenger JD. Decline of childhood *Haemophilus influenzae* type b (Hib) disease in the Hib vaccine era. JAMA 1993;269:221-6.
2. Peltola H, Kilpi T, Antilla M. Rapid disappearance of *Haemophilus influenzae* type b meningitis after routine childhood immunisation with conjugate vaccines. Lancet 1992;340:592-4.
3. Herceg A, Oliver G, Andrews G, Curran M, Crerar S, Andrews R, Evans D. Annual report of the National Notifiable Diseases Surveillance System, 1995. Communicable Diseases Intelligence 1996;20:451.
4. Alho OP, Jokinen K, Pirila T, Ilo A, Oja H. Acute epiglottitis and infant conjugate *Haemophilus influenzae* type b vaccination in northern Finland. Arch Otolaryngol Head Neck Surg 1995;121:898-902.
5. Farley MM, Stephens DS, Brachman PS, Jr., Harvey RC, Smith JD, Wenger JD. Invasive *Haemophilus influenzae* disease in adults. A prospective, population-based surveillance. CDC Meningitis Surveillance Group [see comments]. Ann Intern Med 1992;116:806-12.
6. Casadevall A, Dobroszycki J, Small C, Pirofski LA. *Haemophilus influenzae* type b bacteremia in adults with AIDS and at risk for AIDS [see comments]. Am J Med 1992;92:587-90.

7. Smith PF, Stricof RL, Shayegani M, Morse DL. Cluster of *Haemophilus influenzae* type b infections in adults. JAMA 1988;260:1446-9.
8. Patterson J, Madden GM, Krisiunas EP, Masecar B, Hierholzer WJ Jr., Zervos NJ, Lyons RW. A nosocomial outbreak of ampicillin-resistant *Haemophilus influenzae* type b in a geriatric unit. J Infect Dis 1988;1002-7.
9. Howard AJ, Owens D, Musser JM. Cross-infection due to *Haemophilus influenzae* type b in adults. J Hosp Infect 1991;19:70-2.
10. Frantz TD, Rasgon BM. Acute epiglottitis: Changing epidemiologic patterns. Otolaryngol Head Neck Surg 1993;109:457-60.
11. Mayo-Smith MF, Spinale JW, Donskey CJ, Yukawa M. Acute epiglottitis—an 18 year experience in Rhode Island. Chest 1995;108:1640-7.
12. Berg S, Nylen O, Hugosson S, Prellner K, Carenfelt C. Incidence, aetiology, and prognosis of acute epiglottitis in children and adults in Sweden. Scand J Infect Dis 1996;28:261-4.
13. Barbour ML, Mayon-White RT, Coles C, Crook DWM, Moxon ER. The impact of conjugate vacine on carriage of *Haemophlilus influenzae* type b. J Infect Dis 1995;171:93-8.
